# Identifying distinct trajectories of change in anhedonia during psychological treatment for depression

**DOI:** 10.1017/S0033291726104875

**Published:** 2026-07-07

**Authors:** Daniel Pugh, Rob Saunders, Abbeygail Jones, Barnaby D. Dunn, Jae Won Suh, Joshua Stott, Jon Wheatley, Stephen Pilling, Joshua E. J. Buckman

**Affiliations:** 1 University College London, UK; 2 University of Exeter, UK; 3 Homerton Healthcare NHS Foundation Trust, UK

**Keywords:** anhedonia, depression, epidemiology, growth mixture modeling, mental health outcomes, multinomial logistic regression, NHS talking therapies, psychological therapy, routine outcome measures, statistical methodology, trajectories

## Abstract

**Background:**

While initial anhedonia predicts poor psychotherapy outcomes, little is known about its trajectory during treatment. This study aimed to: (1) identify distinct anhedonia trajectories during high-intensity depression treatment; (2) examine patient and treatment predictors; and (3) compare outcomes across treatment types.

**Methods:**

Sessional anhedonia scores (PHQ-9 item-1) from 22,605 patients in NHS talking therapies (primarily receiving either cognitive-behavioral therapy [CBT] or counseling for depression [CfD]) were analyzed using latent growth curve (LGC) and growth mixture modeling. Multinomial logistic regression examined predictors of class membership.

**Results:**

A quadratic LGC model best fit the data, reflecting a decrease in symptoms before leveling out. Six latent classes emerged. Notably, three “non-responder” classes characterized by linear-stable or minimal-change patterns comprised over 50% of the sample (51.3%). In contrast, two “responder” classes (41.4%) exhibited improvement, typically shifting between sessions 4 and 6. This suggests an early “inflection point” where the trajectory of recovery is established. Poorer response was predicted by unemployment, chronic health conditions, psychotropic medication, and longer wait times. There was only a sufficient sample size to compare CBT and CfD treatment types. While CBT was associated with membership in specific classes, the probability of being a “responder” did not differ significantly between CBT and CfD.

**Conclusions:**

Most patients followed non-responder trajectories, highlighting a major efficacy gap for anhedonia in standard depression protocols. The 4–6 session window suggests that if improvement is not observed early, the treatment strategy may require further evaluation. Further research into targeted anhedonia interventions is essential.

## Introduction

Major depressive disorder is one of the leading causes of disability worldwide and prevalence appears to have increased substantially since the COVID-19 pandemic (Santomauro et al., 2021). Depression is typically defined by two core symptoms (American Psychiatric Association, 2013); low mood or sadness, and anhedonia, the loss of interest or pleasure in most daily activities (often described as feeling ‘flat’ or ‘blunted’ or lacking positive affect; Heininga et al., 2017). Many evidence-based treatments for depression, including the majority of antidepressants and psychological therapies, are thought to particularly target low mood, and it has been argued that they inadequately target anhedonia (Craske et al., 2016; Dunn et al., 2023; Pizzagalli, 2022).

This matters because patients undergoing depression treatment commonly identify amelioration of anhedonia symptoms as their personal priority (Zimmerman et al., 2006). In one study on recovery priorities using a 51-item questionnaire, seven of the ten items ranked most important were related to anhedonia (Demyttenaere et al., 2015). Furthermore, anhedonia is associated with worse treatment outcomes, including poorer psychosocial functioning and longer time to remission (Khazanov et al., 2020; Vinckier, Gourion, & Mouchabac, 2017). Anhedonia symptoms are also more likely to persist following treatment (Alsayednasser et al., 2022; Dunn et al., 2020), and those with residual symptoms after treatment are at greater risk of experiencing relapse or recurrent depressive episodes (Buckman et al., 2018). This raises questions about how anhedonia symptoms evolve during therapy, and for whom current interventions are most effective.

Several recent studies have investigated adapted or novel psychotherapeutic treatments for depression specifically designed to address anhedonia (Craske et al., 2019; Dunn et al., 2019). Although promising, their effectiveness in real-world settings remains variable, with individual differences in symptom progression and treatment response requiring further investigation. In practice, treatment preferences and symptom profiles can shift rapidly, requiring flexible approaches to intervention (Carlier et al., 2019; Fokkema, Smits, Kelderman, & Cuijpers, 2013). Emerging evidence using ecological momentary assessment suggests that anhedonia is highly dynamic, fluctuating over short timescales and exhibiting patterns of instability and change (Gallagher et al., 2025). Understanding of how anhedonia responds over the course of therapy might therefore support development of more tailored and responsive treatment pathways.

Sessional outcome data from large routine datasets (such as those collected in England by NHS Talking Therapies for Anxiety and Depression [TTad]) offer a valuable opportunity to gain that understanding. Symptom trajectories can be modeled over time, including whether changes are consistent across different groups or treatments (Brookes et al., 2001; Ioannidis, 2005). This can clarify when baseline anhedonia severity is a barrier to treatment, and help in the early detection of indicators of poor response. Patients classified as “off-track,” who fail to show expected early improvements, are at heightened risk of non-response or deterioration (Lambert, 2007; Schilling et al., 2021). Recognizing these patterns can help clinicians modify care, such as by intensifying interventions, altering therapeutic approaches, or integrating adjunctive treatments.

Growth Mixture Modeling (GMM) provides a framework to capture this complexity by uncovering distinct symptom trajectories within heterogeneous populations (Saunders, Buckman, & Pilling, 2020a). Researchers can classify patients into distinct change trajectories and examine associations between treatment and those trajectories of change. This improves our understanding of how effective treatments are, who benefits most, and by when improvement might be (Saunders, Buckman, & Pilling, 2020a). Applying this approach to anhedonia can provide novel insights into how responsive current treatments are and how outcomes might be improved when anhedonia is a key barrier to recovery.

### Aims

This study aimed to determine: if there were distinct trajectories of change in anhedonia for people who receive psychological treatments for depression (Aim 1); whether pre-treatment patient characteristics were associated with following different anhedonia trajectories (Aim 2); and whether the type of psychological treatment received impacted the likelihood of following different anhedonia trajectories (Aim 3).

## Method

Analyses were conducted according to a pre-registered protocol (osf.io/s6bjp; Pugh, Buckman, & Saunders, 2024).

### Participants

Participants were adults treated for depression with high-intensity psychological therapies from four of the nine TTad services that contributed data as part of the North and Central East London NHS TTad Service Improvement and Research Network (NCEL NHS TTad SIRN; Saunders et al., 2020b). TTad (formerly IAPT) is a nationally implemented primary care program in England that provides evidence-based psychological therapies, such as CBT and counselling, for common mental health problems. The completion of pre-post-treatment outcomes in TTad is over 99% (Clark, 2018), with most patients completing measures on a sessional basis, providing the opportunity to use trajectory modeling to understand the forms of change in anhedonia symptoms during therapy. Pseudonymized individual patient data were used from patients seen between August 2008 and September 2024.

### Inclusion criteria

- Scored above caseness for depression (i.e. score of 10 or higher on Patient Health Questionnaire-9 [PHQ-9]) at baseline
- Had a problem descriptor (ICD-10 diagnosis that is the agreed focus of treatment) of ‘depression’ or ‘recurrent depression’ (World Health Organization, 2016)
- Aged 18 years or above at referral
- At least three PHQ-9 measurement points taken during high-intensity psychological therapy (within TTad the following therapies are considered “high-intensity”: Cognitive Behavioral Therapy [CBT], Behavioral Activation [BA], Behavioral Couples Therapy, Brief Psychodynamic Psychotherapy, Collaborative Care, Counselling for Depression [CfD/Counselling], Dynamic Interpersonal Therapy [DIT], Interpersonal Psychotherapy [IPT], and Eye Movement Desensitization and Reprocessing [EMDR], Clark, 2018)
- Had individual item data from the PHQ-9

Patients receiving treatment for something other than depression were excluded, as well as patients who received low-intensity treatments only. Individuals who received one session of low-intensity treatment and then three or more sessions of high-intensity treatment were included.

### Measures

#### Anhedonia

The PHQ-9 is a self-report measure of depressive symptoms consisting of 9 items, whereby patients are required to state how often in the last two weeks they were bothered by the items outlined in the questionnaire. The response options are as follows: Not at all (0), Several days (1), More than half the days (2), and Nearly every day (3). This study used item 1, “Little interest or pleasure in doing things,” as a measure of anhedonia.

#### Other measures

In addition, the Generalized Anxiety Disorder Scale (GAD-7) (Spitzer, Kroenke, Williams, & Löwe, 2006) and Work and Social Adjustment Scale (WSAS) (Mundt, Marks, Shear, & Greist, 2002) are self-report measures for generalized anxiety and impaired functioning, which were used to consider the associations between patient characteristics and the different trajectories of change in anhedonia. All domains of the WSAS other than “work” were included at the item level, as this is collinear with employment status.

### Variables

See Table 1 for the list of independent variables for Research Aim 2.

**Table 1.** Independent variables for patient data analysis (Research Aim 2) Table 1. long description.

Category	Variable	Details and options (Notes)
Socio-demographics	Gender	Self-reported at point of referral: Male, Female, Non-binary (Not possible to factor non-binary into analysis due to small sample size, see Appendix A for further details).
	Age	Age at referral (entered as continuous variable)
	Ethnicity	Based on UK census codes (For analysis purposes were grouped into the following categories: White, Mixed, Asian, Black, Other)
	Sexual orientation	Heterosexual, Gay/Lesbian, Bi-sexual, Not Sure (For analysis purposes, patients binarized as following categories: Heterosexual and Non-Heterosexual)
	Index of multiple deprivations (IMD)	Deciles; measures relative deprivation in England. Each area is assigned an overall deprivation score based on combined data, areas are then ranked based on deprivation (Decile 1 representing the 10% most deprived areas and Decile 10 the least).
	Employment status	Derived from self-report data and binarised into Employed/Not Employed categories. “Employed” group included individuals in paid employment (full-time or part-time, employed or self-employed), as well as students in full- or part-time education/training and those engaged in homemaking roles. “Not employed” group comprised individuals who were unemployed and seeking work, long-term sick or disabled, and not in paid work and not seeking work. This approach is consistent with prior research with similar datasets (Buckman et al., 2023)
Psychological factors	Generalized anxiety symptom severity	Assessed using the generalized anxiety disorder scale–7
	Personal functioning	Assessed using work and social adjustment Scale; domains: home management, social activities, private leisure activities, close relationships
Health factors	Long-term health conditions	Status based on presence of any self-reported long-term physical conditions: Yes, No
	Psychotropic medication	Status recorded based on self-report data as Not Taking, Prescribed and Not Taking, and Prescribed and Taking. Binarised for the purposes of this study as Taking and Not taking.
Service factors	Service	Trust 1, Trust 2, Trust 3, Trust 4
	Waiting time	Waiting time between the referral to the services and the first assessment
		Waiting time between the first appointment and second appointment
Cohort factors	Cohort	Year in which patient began treatment (entered as a continuous variable)

To consider the degree to which treatment type was associated with trajectory classes, multinomial regression models were expanded to include treatment type as an independent variable. As patients may receive more than one treatment type, analyses focused on the main treatment received, i.e. identified treatment type for >50% of sessions.

### Statistical analysis

This study used existing sessional anhedonia (PHQ-9 item-1) data from individuals receiving high-intensity psychological treatment. Data from the first 12 sessions of treatment were modeled. A 12-session model was selected to maximize data inclusion without excessive missingness, in line with previous depression trajectory modeling studies using similar datasets, which have typically examined change over 10–13 sessions (Saunders et al., 2019; Skelton et al., 2023).

#### Research Aim 1

To understand if there are distinct trajectories of change in anhedonia, latent growth curve analysis (LGC) first estimated the expected response curve over 12 sessions. The model was tested using linear, quadratic, and cubic factors, following the standard modeling approach using polynomial growth functions and acknowledging that change during psychological therapy is typically non-linear (Saunders et al., 2019). There are no fixed a priori sample size requirements for LGC modeling, however guidance from structural equation modeling literature suggests that samples of approximately 200 or more are typically sufficient for stable estimation, with larger samples recommended for more complex models (Kline, 2016). We expected the present sample to substantially exceed this recommendation. Model fit was assessed using the following metrics: the Comparative Fit Index (CFI), Tucker-Lewis Index (TLI), and Root Mean Squared Error of Approximation (RMSEA). CFI and TLI scores above 0.9 and 0.95 indicate good and excellent fit, whereas RMSEA scores <0.08 and <0.05 indicate reasonable and close/good-fit (Kline, 2016).

Growth mixture odeling (GMM) was then used to identify subgroups based on sessional trajectories of symptom-change (Saunders et al., 2019; Vittengl, Clark, Thase, & Jarrett, 2013). Similarly, for LGC, seeking as large a sample as possible is recommended, with simulation studies recommending larger samples (1000+) as this improves class detection and parameter stability (Kim, 2012; Nylund, Asparouhov, & Muthén, 2007). As there were no specific hypotheses about class number, models were estimated from two classes upwards with fit assessed sequentially. GMM models were compared using the Vuong-Lo-Medell-Rubin Likelihood Ratio Test (VLMR-LRT), Akaike Information Criterion (AIC), and Bayesian Information Criterion (BIC). *P*-values >0.05 indicate the present model does not significantly improve fit over a simpler model with one fewer class, whilst lower AIC and BIC values indicate better fit (Nylund, Asparouhov, & Muthén, 2007). Decisions about the optimal model solution were made in line with best practice. The smallest class should represent at least 5% of the sample to ensure clinical utility (Bauer & Curran, 2003; Spinhoven et al., 2016). When in conflict with other indices of model fit (including VLMR-LRT and AIC), the BIC is generally prioritized when dealing with large sample sizes due to greater penalization of model complexity (Chen et al., 2017; Sen & Cohen, 2024), consistency in model selection (Vrieze, 2012), and robust performance across different simulation scenarios (McNeish & Harring, 2017).

There are no universally agreed a priori sample size requirements for LGC or GMM; seeking as large a sample is typically recommended, however, for both approaches. Guidance from structural equation modeling literature suggests approximately 200 or more are needed for stable estimation in LGC, and greater needed for more complex models (Kline, 2016), while simulation studies in mixture modeling suggest that larger samples (1000+) improve class detection and parameter stability, especially if a large number of classes might be identified (Kim, 2012; Nylund, Asparouhov, & Muthén, 2007). All available cases were therefore sought, and we expected the present sample to substantially exceed these recommendations.

#### Research Aim 2

R3STEP was used to examine the associations between baseline patient characteristics and anhedonia trajectory class membership. R3STEP is a three-step Mplus procedure that estimates the latent class model, then assigns individuals to classes while accounting for classification uncertainty, and finally uses class assignments in a multinomial logistic regression wherein all predictors are entered simultaneously (Asparouhov & Muthén, 2014). This retains variance in class membership likelihood and reduces bias in estimating associations relative to conducting analyses in separate steps.

#### Research Aim 3

For the third research question, examining the association between treatment type and trajectory class membership, R3STEP was also used. To ensure statistical power and reliable estimates for logistic regression (Bujang, Sa’at, Tg Abu Bakar Sidik, & Chien Joo, 2018), only treatment types with a sufficient sample size, as determined by the formula *n*=100 + 50(*𝑖*), were included (*𝑖* referring to the number of independent variables in the model). Only CfD and CBT, therefore, were compared, as the third most popular treatment type, IPT (*n*=223), did not meet the necessary participant threshold of *n* = 250.

### Missing data, software, and packages

LGC and GMM modeling was performed using Mplus(v8). Missing sessional PHQ-9 item −1 data was handled using Full Information Maximum Likelihood (FIML) as standard (Geiser, 2012). Multiple imputation was also used for handling missing data for the independent variables listed in Table 1 (Asparouhov & Muthén, 2022).

## Results

### Descriptive statistics

A total of 22,605 patients met the inclusion criteria and attended an average of 10.32 sessions. Table 2 presents baseline characteristics and demographic data.

**Table 2.** Baseline characteristics and demographic data Table 2. long description.

Characteristic	Category	*n*	%
Therapeutic treatment	CBT	18615	82.34
	Counselling for depression	3410	15.09
	Other/Missing*	580	2.57
Gender	Female	15475	68.46
	Male	7027	31.09
	Nonbinary	33	0.15
	Missing	70	0.31
Sexuality	Heterosexual	16586	73.37
	Gay/Lesbian	728	3.22
	Bisexual	669	2.96
	Not sure	317	1.40
	Missing/declined to answer	4305	19.04
Ethnicity	White	13552	59.95
	Mixed	1515	6.70
	Asian	3055	13.51
	Black	2657	11.75
	Other	1081	4.78
	Missing	745	3.30
Religion	No religion	5644	24.97
	Christian	4277	18.92
	Muslim	2048	9.06
	Jewish	503	2.23
	Hindu	496	2.19
	Other	919	4.07
	Missing	8718	38.57
Employment status	Employed	15947	70.55
	Unemployed	5898	26.09
	Missing	760	3.36
Taking medication	No	12574	55.62
	Yes	8350	36.94
	Missing	1681	7.44
1 = < Long-term condition	Yes	6143	27.18
	No	10177	45.02
	Missing	6,285	27.80
Service	Trust 1	8392	37.12
	Trust 2	4620	20.44
	Trust 3	4776	21.13
	Trust 4	4817	21.31
**Characteristic**	***n***	**Mean**	**SD**
Age at referral	22605	38.42	13.22
PHQ–9 score	22553	16.44	5.70
GAD–7 score	22545	13.52	5.02
WSAS score	22037	20.36	9.01
Weeks between referral and assessment	22600	4.63	7.59
Weeks between referral and treatment	21793	13.35	11.05
Number of attended sessions	19124	10.33	4.92

*Note:* CBT, Cognitive Behavioral Therapy; PHQ-9, Patient Health Questionnaire-9; GAD-7, Generalized Anxiety Disorder Scale-7; WSAS, Work and Social Adjustment Scale. All column headers in each section of the table are bolded.

*No specified therapeutic treatment other than CBT or Counselling was delivered to >1% of participants (Interpersonal Therapy/IPT was the third-most delivered, to 223 participants/0.98% of the sample), so all other treatments were grouped into the “Other” category.

### Research Aim 1

#### Latent growth curve modeling

**Figure 1.**
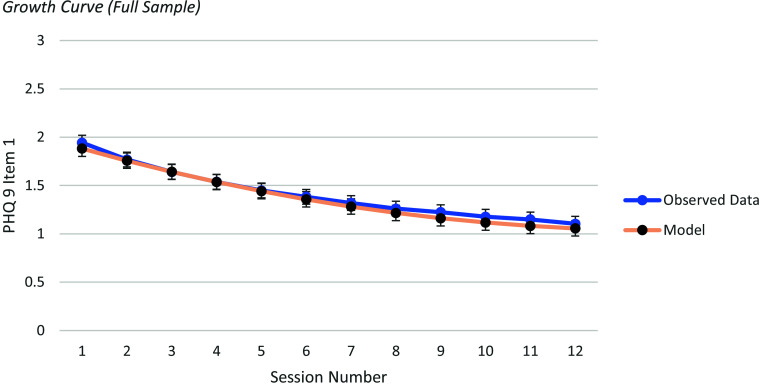
Growth curve (full sample). Figure 1. long description.

To understand if there are distinct trajectories of change in anhedonia, the first best fit for the data was obtained using a quadratic model, which is presented in Figure 1, displaying a decrease in anhedonia scores before levelling out. Fit and growth parameter statistics are displayed in Appendix A.

#### Growth mixture modeling

**Figure 2.**
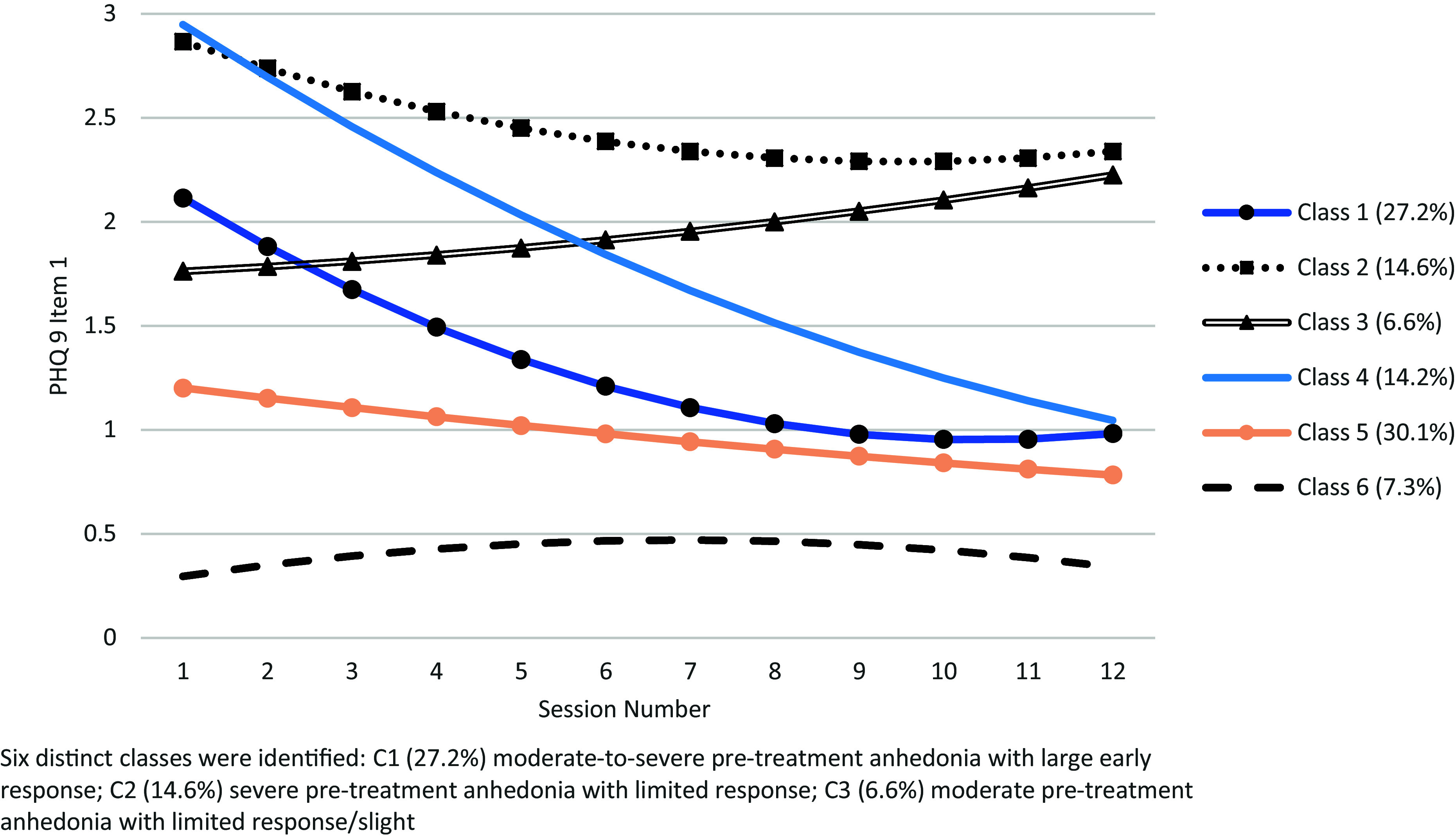
Trajectory classes of anhedonia over time. Figure 2. long description.

GMM was performed on anhedonia scores using quadratic models, with a six-class solution preferred (model fit statistics in Appendix B). The identified trajectories are displayed in Figure 2, with class labels assigned based on visual interpretation of trajectory shape intended as descriptive summaries rather than formal classifications of change, with growth parameter statistics presented in Appendix B.

### Research Aim 2

#### Baseline variables associated with trajectories

R3STEP was used for multinomial regression models that explored associations between baseline characteristics and different trajectories of anhedonia change. See Table 3 for odds ratios (OR), 95% confidence intervals (CI), and *p*-values for the different classes compared against C5. This was pragmatically selected as the reference group because it was the largest class representing the modal pattern of change for the sample (mild baseline symptoms and little response to treatment).

**Table 3.** Associations between baseline characteristics and anhedonia trajectory classes 1, 2, 3, 4, and 6 relative to class 5 (mild baseline anhedonia, little response) Table 3. long description.

Research Aim 2					
Baseline predictor	C#1 (moderate baseline, responder)	C#2 (severe baseline, non-responder)	C#3 (moderate baseline, non-responder)	C#4 (severe baseline, responder)	C#6 (little-no baseline anhedonia symptoms)
Referral year	**1.005 (1.003–1.007), *p* < 0.001**	**0.924 (0.921–0.928), *p* < 0.001**	**0.948 (0.943–0.952), *p* < 0.001**	**1.009 (1.006–1.012), *p* < 0.001**	**0.956 (0.952–0.959), *p* < 0.001**
Gender					
Female	1.0	1.0	1.0	1.0	1.0
Male	**0.941 (0.930–0.951), *p* < 0.001**	**0.807 (0.794–0.822), *p* < 0.001**	**0.906 (0.883–0.929), *p* < 0.001**	**0.774 (0.765–0.783), *p* < 0.001**	**1.381 (1.355–1.410), *p* < 0.001**
Sexual orientation					
Heterosexual	1.0	1.0	1.0	1.0	1.0
Non-heterosexual	1.067 (0.988–1.152), *p* = 0.079	**0.673 (0.570–0.795), *p* = 0.002**	1.117 (0.939–1.330), *p* = 0.166	**1.093 (1.002–1.192), *p* = 0.030**	**1.259 (1.096–1.447), *p* < 0.001**
GAD7	**1.093 (1.080–1.106), *p* < 0.001**	**1.321 (1.287–1.355), *p* < 0.001**	**1.117 (1.092–1.143), *p* < 0.001**	**1.181 (1.157–1.203), *p* < 0.001**	**0.895 (0.878–0.912), *p* < 0.001**
WSAS2	**1.055 (1.027–1.083), *p* < 0.001**	**1.179 (1.134–1.227), *p* < 0.001**	**1.099 (1.044–1.156), *p* < 0.001**	**1.114 (1.076–1.151), *p* < 0.001**	**0.796 (0.789–0.804), *p* < 0.001**
WSAS3	**1.135 (1.104–1.167), *p* < 0.001**	**1.321 (1.253–1.391), *p* < 0.001**	**1.138 (1.129–1.148), *p* < 0.001**	**1.186 (1.142–1.233), *p* < 0.001**	**0.890 (0.848–0.934), *p* < 0.001**
WSAS4	**1.068 (1.041–1.098), *p* < 0.001**	**1.178 (1.131–1.225), *p* < 0.001**	**1.086 (1.076–1.096), *p* < 0.001**	**1.142 (1.105–1.181), *p* < 0.001**	**0.897 (0.890–0.904), *p* < 0.001**
WSAS5	0.982 (0.958–1.007), *p* = 0.17	**1.056 (1.015–1.098), *p* = 0.004**	**1.036 (1.004–1.070), *p* = 0.026**	**1.032 (0.998–1.066), *p* = 0.049**	**0.926 (0.887–0.965), *p* < 0.001**
Waiting time (from referral to assessment)	0.991 (0.982–1.001), *p* = 0.073	0.994 (0.986–1.002), *p* = 0.135	0.997 (0.986–1.008), *p* = 0.618	**0.979 (0.967–0.991), *p* = 0.003**	1.000 (0.991–1.009), *p* = 1.0
Waiting time (until second appointment)	0.997 (0.992–1.002), *p* = 0.318	**1.007 (1.000–1.013), *p* = 0.019**	1.007 (0.999–1.015), *p* = 0.079	**0.992 (0.985–0.998), *p* = 0.008**	0.997 (0.989–1.005), *p* = 0.454
Age	**1.007 (1.003–1.012), *p* < 0.001**	**1.026 (1.019–1.032), *p* < 0.001**	1.004 (0.996–1.013), *p* = 0.423	**1.015 (1.010–1.021), *p* < 0.001**	1.002 (0.996–1.008), *p* = 0.505
Medication					
Not taking	1.0	1.0	1.0	1.0	1.0
Taking	**1.214 (1.175–1.253), *p* < 0.001**	**1.767 (1.689–1.848), *p* < 0.001**	**1.642 (1.541–1.748), *p* < 0.001**	**1.631 (1.575–1.692), *p* < 0.001**	**0.727 (0.684–0.773), *p* < 0.001**
Long-term health condition(*s*)					
Yes	0.982 (0.917–1.054), *p* = 0.62	**1.393 (1.282–1.513), *p* < 0.001**	**1.462 (1.271–1.684), *p* < 0.001**	1.060 (0.979–1.148), *p* = 0.122	**0.850 (0.760–0.950), *p* = 0.016**
No	1.0	1.0	1.0	1.0	1.0
Employment					
Employed	1.0	1.0	1.0	1.0	1.0
Unemployed	**1.292 (1.252–1.335), *p* < 0.001**	**5.025 (4.878–5.181), *p* < 0.001**	**3.546 (3.390–3.717), *p* < 0.001**	**1.730 (1.678–1.786), *p* < 0.001**	**0.621 (0.577–0.668), *p* < 0.001**
IMD Decile	0.987 (0.962–1.011), *p* = 0.320	**0.912 (0.880–0.947), *p* < 0.001**	**0.923 (0.914–0.931), *p* < 0.001**	**0.948 (0.919–0.977), *p* < 0.001**	**1.065 (1.029–1.103), *p* < 0.001**
Ethnicity					
White	0	0	0	0	0
Black	**1.129 (1.096–1.161), *p* < 0.001**	**0.776 (0.735–0.819), *p* < 0.001**	**1.174 (1.104–1.247), *p* < 0.001**	**1.399 (1.344–1.458), *p* < 0.001**	**0.690 (0.644–0.740), *p* < 0.001**
Mixed	1.020 (0.983–1.058), *p* = 0.262	**0.835 (0.775–0.901), *p* < 0.001**	**0.865 (0.785–0.954), *p* = 0.012**	**1.160 (1.105–1.218), *p* < 0.001**	**0.872 (0.809–0.939), *p* = 0.002**
Asian	**1.239 (1.200–1.280), *p* < 0.001**	**1.314 (1.261–1.370), *p* < 0.001**	**1.524 (1.435–1.621), *p* < 0.001**	**1.456 (1.389–1.524), *p* < 0.001**	**0.868 (0.816–0.923), *p* < 0.001**
Other	**0.843 (0.797–0.891), *p* < 0.001**	**1.202 (1.133–1.276), *p* < 0.001**	**1.988 (1.866–2.114), *p* < 0.001**	**1.443 (1.364–1.527), *p* < 0.001**	**0.698 (0.631–0.773), *p* < 0.001**
Service					
Trust 1	0	0	0	0	0
Trust 2	**0.833 (0.818–0.848), *p* < 0.001**	**0.966 (0.941–0.993), *p* = 0.014**	**1.161 (1.120–1.203), *p* < 0.001**	**0.706 (0.692–0.720), *p* < 0.001**	**0.915 (0.886–0.944), *p* < 0.001**
Trust 3	**0.938 (0.927–0.949), *p* < 0.001**	**1.131 (1.105–1.159), *p* < 0.001**	**1.091 (1.059–1.122), *p* < 0.001**	**0.653 (0.642–0.664), *p* < 0.001**	**1.587 (1.553–1.623), *p* < 0.001**
Trust 4	**1.080 (1.071–1.088), *p* < 0.001**	1.012 (0.995–1.031), *p* = 0.18	**0.967 (0.945–0.990), *p* = 0.005**	**0.793 (0.781–0.805), *p* < 0.001**	**2.198 (2.160–2.237), *p* < 0.001**
Research Aim 3					
Baseline predictor	C#1 (Moderate baseline, responder)	C#2 (Severe baseline, non-responder)	C#3 (Moderate baseline, non-responder)	C#4 (Severe baseline, responder)	C#6 (Little-no baseline anhedonia symptoms)
Treatment type					
CBT	**1.183 (1.019–1.374), *p* = 0.028**	**1.252 (1.061–1.477), *p* = 0.008**	1.083 (0.813–1.444), *p* = 0.585	**1.317 (1.091–1.591), *p* = 0.004**	0.859 (0.083–0.711), *p* = 0.117
Counseling for depression	1.0	1.0	1.0	1.0	1.0

*Note:* All significant results (*p* < 0.05) in bold. 580 participants excluded for Research Aim 3.

Participants with the following baseline characteristics were more likely to belong to trajectory classes with typically more severe baseline anhedonia than C5 (C1,2,3,4):

- Male
- Older age (non-significant C3)
- Comorbid long-term health condition (non-significant C1,3)
- Unemployed
- Higher anxiety
- Higher functional impairment across the 4 WSAS items (C1 WSAS5/Relationships non-significant)
- Lower IMD Decile (non-significant C1)
- Not taking medication
- Asian ethnicity (compared to White ethnicity)

These characteristics also reduced the likelihood of being in C6 (no anhedonia symptoms) except for age (non-significant). There were conflicting directions of significant effects across different severities for the remaining predictors (see Table 4 for simplified results).

**Table 4.** Simplified table describing associations between baseline characteristics and anhedonia trajectory classes 1, 2, 3, 4, and 6 relative to 5 (Mild Baseline Anhedonia, little response) Table 4. long description.

Baseline predictor	C#1 (Moderate baseline, responder)	C#2 (Severe baseline, non-responder)	C#3 (Moderate baseline, non-responder)	C#4 (Severe baseline, responder)	C#6 (Little-no baseline anhedonia symptoms)
Referral year: Higher	+	−	−	−	−
Gender: male	+	+	+	+	−
Sexual orientation: Non-heterosexual		+		−	−
GAD7: higher	+	+	+	+	−
WSAS2/home management: higher	+	+	+	+	−
WSAS3/social leisure: higher	+	+	+	+	−
WSAS4/private leisure: higher	+	+	+	+	−
WSAS5/relationships: higher		+	+	+	−
Waiting time to assessment: higher				−	
Waiting time to second appointment: higher		+		−	
Age: Older	+	+		+	
Medication: taking	−	−	−	−	+
Long term condition: yes		+	+		−
Employment: unemployed	+	+	+	+	−
IMD decile: lower		+	+	+	−
Ethnicity: black	+	−	+	+	−
Ethnicity: mixed		−	−	+	−
Ethnicity: Asian	+	+	+	+	−
Ethnicity: Other	−	+	+	+	−
Service: Trust 2	−	−	+	−	−
Service: Trust 3	−	+	+	−	+
Service: Trust 4	+		−	−	+
Treatment type: CBT	+	+		+	

*Note: Key: + increased likelihood – reduced likelihood.*

#### Responder versus non-responder comparisons

Comparisons were also made between responder and non-responder classes with similar baseline anhedonia levels.

Severe baseline comparisons C4 (responders) and C2 (non-responders) for participants with severe-baseline anhedonia were compared (see Table 5).

**Table 5.** Associations between baseline characteristics and anhedonia trajectory for class 4 (Responders, severe baseline anhedonia) relative to class 2 (Non-responders, severe baseline anhedonia) Table 5. long description.

Research Aim 2	
Baseline predictor	Class 4 (OR, 95% CI, *p*-value)
Referral year	**1.092 (1.087–1.096), *p* < 0.001**
Gender	
Female	1.0
Male	**0.958 (0.939–0.978), *p* < 0.001**
Sexual orientation	
Heterosexual	1.0
Non-heterosexual	**1.624 (1.357–1.943), *p* = 0.001**
GAD7	**0.894 (0.862–0.927), *p* < 0.001**
WSAS2	**0.944 (0.896–0.994), *p* = 0.021**
WSAS3	**0.899 (0.835–0.967), *p* = 0.001**
WSAS4	0.969 (0.918–1.024), *p* = 0.243
WSAS5	0.977 (0.927–1.031), *p* = 0.371
Waiting time (from referral to assessment)	0.985 (0.970–1.000), *p* = 0.059
Waiting time (until second appointment)	**0.985 (0.977–0.994), *p* = 0.003**
Age	**0.990 (0.982–0.999), *p* = 0.012**
Medication	
Not taking	1.0
Taking	**0.924 (0.877–0.974), *p* = 0.002**
Long-term health condition(s)	
Yes	**0.762 (0.684–0.848), *p* < 0.001**
No	1.0
Employment	
Employed	1.0
Unemployed	**0.344 (0.330–0.358), *p* < 0.001**
IMD decile	1.038 (0.988–1.091), *p* = 0.151
Ethnicity	
White	0
Black	**1.804 (1.692–1.922), *p* < 0.001**
Mixed	**1.389 (1.275–1.514), *p* < 0.001**
Asian	**1.107 (1.046–1.172), *p* = 0.001**
Other	**1.201 (1.108–1.302), *p* < 0.001**
Service	
Trust 1	0
Trust 2	**0.730 (0.710–0.751), *p* < 0.001**
Trust 3	**0.577 (0.560–0.595), *p* < 0.001**
Trust 4	**0.783 (0.764–0.802), *p* < 0.001**
Research Aim 3	
Baseline predictor	Class 4 (OR, 95% CI, *p*-value)
Treatment type	
CBT	0.950 (0.724–1.247), *p* = 0.712
Counselling for depression	1.0

*Note:* Column headers are bolded, as are significant effects (*p* ≤ 0.05).

Participants in the responder class were more likely to have the following characteristics (all categories not mentioned were non-significant):

- Male
- Younger
- Non-heterosexual
- Recent cohort
- Shorter wait to second appointment
- Lower anxiety
- Less functional impairment (WSAS-2&3 only)
- Employed
- Not taking medication
- No long-term health conditions
- Be of any non-White ethnicity
- Treated at Trust-1

Moderate baseline comparisons C1 (responders) and C3 (non-responders) were compared for patients with moderate-baseline anhedonia (see Table 6).

**Table 6.** Associations between baseline characteristics and anhedonia trajectory for class 1 (responders, moderate baseline anhedonia) relative to class 3 (non-responders, moderate baseline anhedonia) Table 6. long description.

Research Aim 2	
Baseline predictor	Class 3 (OR, 95% CI, *p*-value)
Referral year	**1.061 (1.056–1.066), *p* < 0.001**
Gender	
Female	1.0
Male	**1.039 (1.010–1.070), *p* = 0.011**
Sexual orientation	
Heterosexual	1.0
Non-heterosexual	0.955 (0.785–1.160), *p* = 0.628
GAD7	0.979 (0.955–1.003), *p* = 0.077
WSAS2	0.960 (0.908–1.016), *p* = 0.145
WSAS3	0.997 (0.969–1.027), *p* = 0.841
WSAS4	0.984 (0.957–1.012), *p* = 0.249
WSAS5	**0.948 (0.911–0.986), *p* = 0.005**
Waiting time (from referral to assessment)	0.995 (0.980–1.010), *p* = 0.531
Waiting time (until second appointment)	**0.990 (0.981–0.999), *p* = 0.012**
Age	1.003 (0.993–1.013), *p* = 0.549
Medication	
Not taking	1.0
Taking	**0.739 (0.689–0.793), *p* < 0.001**
Long-term health condition(s)	
Yes	**0.672 (0.580–0.778), *p* < 0.001**
No	1.0
Employment	
Employed	1.0
Unemployed	**0.364 (0.346–0.384), *p* < 0.001**
IMD decile	**1.070 (1.042–1.098), *p* < 0.001**
Ethnicity	
White	0
Black	0.962 (0.907–1.020), *p* = 0.182
Mixed	**1.179 (1.066–1.304), *p* = 0.007**
Asian	**0.813 (0.762–0.869), *p* < 0.001**
Other	**0.424 (0.392–0.459), *p* < 0.001**
Service	
Trust 1	0
Trust 2	**0.717 (0.688–0.747), *p* < 0.001**
Trust 3	**0.860 (0.835–0.886), *p* < 0.001**
Trust 4	**1.116 (1.088–1.143), *p* < 0.001**
**Research Aim 3**	
Baseline predictor	Class 3 (OR, 95% CI, *p*-value)
Treatment type	
CBT	0.916 (0.664–1.264), *p* = 0.594
Counselling for depression	1.0

*Note:* All significant results (*p* < 0.05) in bold. 580 participants excluded for Research Aim 3.

Participants in the responder class were more likely to have the following characteristics:

- Female
- Recent cohort
- Shorter wait to second appointment
- Less functional impairment on WSAS5/Close relationships
- Employed
- Not taking medication
- No long-term health conditions
- Higher IMD-Decile
- Mixed-ethnicity (Asian- or Other-ethnicity less likely)
- Treated at Trust-4 (Trust-2/3 less likely)

### Research Aim 3

Research Aim 3 compared CBT and Counseling for Depression (CfD). There was insufficient power in the dataset for a third treatment type to be considered due to low numbers (Based on the *n* = 100 + 50(*𝑖*) method, a minimum of 250 participants were required for any further treatment types to be considered). The small number of participants who received these were excluded from this specific analysis.

Participants who received CBT were, relative to class 5, more likely to be members of classes 1, 2, or 4 (see Table 3).

The probability of being in either of the responder classes when compared to non-responder classes was not affected by receipt of Counselling or CBT (see Tables 5 and 6).

## Discussion

This study used routine treatment data to identify distinct trajectories of anhedonia change during high-intensity psychological therapy for depression, and examined factors associated with those trajectories. With regard to research aim one, six trajectories were identified. Only patients in two classes (41.4% of the cohort) showed significant improvement, with positive change evident in PHQ-9 Item-1 by session 6. This suggests that when anhedonia responded to treatment, change was evident by the midpoint of therapy. This aligns with evidence that much symptom change during CBT for depression occurs in the first six sessions (Abel, Hayes, Henley, & Kuyken, 2016; Tang & DeRubeis, 1999), and extends that pattern to anhedonia specifically.

Compared with previous trajectory modeling research on overall depression symptom severity, these findings suggest anhedonia may respond differently to treatment. Two large-scale studies identified four trajectories of PHQ-9 total scores, the first using an older, smaller NCEL NHS TTad SIRN dataset from one Trust (Saunders et al., 2019), and the latter replicating those results using a South London NHS TTad dataset (Skelton et al., 2023). The current study identified qualitatively different trajectories, with only classes 2 (severe baseline-fast improvement) and 4 (severe baseline-limited improvement) having direct equivalents. Class 6, characterized by little-to-no symptoms throughout treatment, had no analogue in studies for depression (or anxiety). Given that participants were included for depression, rather than anhedonia, this class potentially reflects people who do not experience anhedonia as part of their depression.

For the second research question, clearly identified risk factors for non-response were unemployment, long-term health conditions, antidepressant medication use, and experiencing a longer wait until the second appointment. Participants who received treatment more recently were also more likely to be placed in responder trajectory classes.

Unemployment, being the most significant risk factor, was broadly unsurprising given Buckman and colleagues found a very strong effect of unemployment on poor treatment prognosis for depression, regardless of treatment type (2022). Associations for both antidepressant use and long-term health conditions similarly align with existing evidence (Buckman et al., 2021a). One would have expected from the existing evidence base that patients who are from more deprived areas, and suffering from more comorbidity with regard to both anxiety and functional impairment to be consistently associated with non-responding trajectories (Prieto-Vila et al., 2024; Saunders, Buckman, & Pilling, 2020a; Skelton et al., 2023), however these were only associated with one of two non-responding trajectories.

The consistent small effect of time matches existing research around treatment outcomes in England, as services have shown modest improvements in outcomes year-on-year, reflective of developments in clinical practice (Clark, 2018; Clark et al., 2018; Saunders et al., 2020b).

There was conflicting information on ethnicity, with no individual ethnic group consistently associated with responding or non-responding trajectory classes across both moderate-baseline and severe-baseline comparisons, except for mixed-ethnicity patients, who appeared consistently associated with response. The mechanism behind this is unclear, especially given that this has not been reported across wider depression or anxiety literature (and should be interpreted cautiously). There was also an association between waiting time to the second appointment (often considered the effective start of treatment in TTad) and non-responder class membership, consistent with previous research using TTad data found that waiting time for treatment was negatively associated with reliable recovery (Clark et al., 2018).

Most notably, no trajectory class with baseline anhedonia showed complete elimination by endpoint. This supports evidence that anhedonia symptoms are associated with a greater chance of residual symptoms following therapeutic treatment (Alsayednasser et al., 2022). This is particularly problematic because the experience of residual symptoms post-treatment is one of the strongest risk factors for experiencing a relapse of depression (Buckman et al., 2018; Judd, Paulus, & Zeller, 1999). Furthermore, 51.3% were more likely to follow trajectories indicating little to no anhedonia response. Although this limited response rate is broadly similar to that seen for psychotherapeutic depression treatment outcomes (Cuijpers et al., 2014, 2021), anhedonia may require particular attention because it may persist even among patients otherwise responding to treatment. Since symptom change was typically evident by session six, this highlights the importance of identifying and targeting anhedonia early in treatment. A strong recommendation, therefore, would be a need for future therapeutic options to better prioritize and optimize anhedonia treatment.

### Limitations

Although predictors were examined within a single multivariable model, reducing concerns associated with multiple independent testing, the inclusion of multiple predictors nonetheless introduces a potential risk of Type I error inflation. As such, findings should be interpreted with consideration of effect sizes, confidence intervals, and overall patterns rather than isolated *p*-values. Class labels were assigned based on visual interpretation of trajectory shape rather than formal thresholds (e.g. reliable change indices), which may introduce subjectivity in their interpretation.

The PHQ-9 presents several limitations. It includes only one anhedonia item, scored 0 (Not at all), 1 (Several days), 2 (More than half of the days), or 3 (Nearly every day). This limited range constrains variability in the data, making capturing subtle differences more difficult, which potentially caps the anhedonia-score distribution. Furthermore, the wording of the PHQ-9 may introduce measurement error; the scale is used to consider the severity of the respondent’s depressive symptoms, yet response options capture symptom frequency. A participant may experience anhedonia as severely problematic or crippling, but not frequently enough to merit “More than half of the days,” leading them to score 1. Similarly, the gap between “Not at all” and “Several days” may force participants into an imprecise response.

Due to the absence of a controlled study design, confounding remains a potential issue. As with other studies using data from the NHS TTad datasets (Saunders et al., 2019), there is no information on treatment protocol adherence within CBT or CfD sessions, and no information on the criteria used to allocate patients to treatment type. There may be confounding from undiagnosed or unmeasured comorbidities and other factors associated with the lack of improvement in anhedonia symptoms. In particular, this applies to our self-report measure on psychotropic medication use and long-term conditions. For example, people who were taking medication could have stopped taking it during the course of treatment, and anhedonic symptoms are a symptom of antidepressant withdrawal (Moncrieff, Read, & Horowitz, 2024). More broadly, it did not capture medication type, class, dosage, or duration of use, which may all have differential impacts on outcomes. Similarly, the presence of a “long-term condition” was derived from routinely collected data and based on self-report without a standardized definition of duration or severity. Lastly, several additional characteristics linked with treatment prognosis were unavailable, including level of previous treatment/therapy experience, depression chronicity, marital status, social support, and personality disorder comorbidity (Buckman et al., 2021a, 2021b, 2021c, 2022; DeRubeis et al., 2014).

Reverse causality may also limit the interpretation of some associations. While one explanation is that taking medication may reduce the effectiveness of psychotherapy on anhedonia symptoms, alternatively, people on medication prior to starting psychotherapy may have experienced more treatment-resistant depression, as those who had responded may not have needed therapeutic support. Supporting this, medication use was a predictor of lower baseline severity and non-response among those with higher baseline severity. Similarly, there is a clear association between unemployment and poorer outcomes, but it is equally possible that unemployment leads to more treatment-resistant anhedonia, as those suffering from severe anhedonia and depression are less likely to be employed (Rizvi et al., 2015; Vinckier, Gourion, & Mouchabac, 2017).

Selection biases could also limit generalizability. Firstly, high-intensity only interventions may overrepresent those with chronic or treatment-resistant depression. Attritional bias is also possible as those who dropped out before taking three measurement points may systemically differ, and experience more severe anhedonia. Finally, the sample was drawn from four services flowing data to the NCEL NHS TTad SIRN; demographically representative of these London-based services, potentially limiting generalizability to non-urban populations or other urban locations outside London. This, however, presents significant strengths. The sample is more ethnically diverse than NHS TTad nationally (NHS Digital, 2024), which is important given the need to avoid perpetuating systematic biases by under-representing historically marginalized groups. Greater representation of smaller groups contributes to a more inclusive and equitable evidence base and enhances the potential for identifying important subgroup effects and generalizing findings to those smaller groups in the wider population. Furthermore, the primary measure for this study, the PHQ-9, has shown across a range of communities to have good reliability and validity (Beck, Naz, Brooks, & Jankowska, 2019).

Finally, 97.5% of patients received either CBT or Counseling, narrowing the ability to make conclusions about the full treatment landscape. Despite this, homogeneity in the data emerged naturally and it remains a reasonable approximation of the current breadth of high-intensity treatments in England, given that last year, 93.3% of high-intensity therapy courses in TTad services were either CBT or Counselling (NHS Digital, 2024).

### Implications

Firstly, there were notable associations between trajectories of anhedonia and patient characteristics. Clinicians may be able to gauge anhedonia symptom response by the midpoint of therapy. As such, therapists should track anhedonia from the start of treatment to support timely assessment and adjustment of the care plan. For those following responder trajectories, change was typically evident around session six. Early improvement can be seen as a positive prognostic indicator, reinforcing the continuation of the current treatment strategy. These patients could also potentially be considered for less frequent reviews to save resources.

Those showing no signs of improvement by session six could be monitored closer and more regularly with a view to potentially adapting their care plan. This may prompt discussions around additional personal or environmental factors in their lives, including possible comorbid conditions. More regular reviews may need to be considered, alongside more intensive treatment options, or potentially contacting their listed General Practitioner for alternative means of medical management.

This study also indicates several risk factors for non-response, allowing for the earlier identification of patients more likely to show limited improvement in anhedonia symptoms. For example, an individual with severe anhedonia with several non-responder risk factors (e.g. unemployed, taking medication, long-term health conditions) may require a more intensive treatment approach. While it may be useful for some patients to consider pivoting away from CBT and counselling, there were insufficient data to provide more specific recommendations. One possible option may be more targeted approaches that specifically address anhedonia, such as Augmented Depression Therapy/ADepT (Dunn et al., 2019, 2023), although the evidence for this approach comes only from pilot RCT data and a more robust investigation of the effects is warranted before it can be considered for those with anhedonia more widely.

The presence of residual symptoms following treatment for depression is a significant risk factor for relapse and recurrence (Buckman et al., 2018). It is notable, therefore, that this study indicates a substantial proportion of patients exhibit little improvement in anhedonia symptoms following an episode of high-intensity psychological treatment such as CBT or Counseling. One option may be to consider prolonging treatment for those with limited response, though results from this study do not necessarily indicate that to be beneficial. For those patients exhibiting strong residual anhedonia, they could be recommended for additional follow-ups to monitor the risk of relapse, or relapse prevention interventions, including Collaborative Care (Moriarty et al., 2020), Continuation-CT/CBT or Mindfulness-based CBT (Bockting et al., 2015; Kuyken et al., 2016).

Certain factors were consistently tied to a higher risk of non-response, specifically unemployment and long-term health conditions. One possible explanation for this may be the extent to which participants are able to engage in activities in which they enjoy or find a sense of purpose or achievement is reduced. This reduced engagement creates a cycle by which the lack of pleasurable experiences might reduce positive affect and reinforce feelings of apathy and demotivation (Szczepanik et al., 2017).

Given that unemployment was by far the most significant risk factor for non-response, concurrent employment support programs may be particularly valuable for these patients, a comparison of which might be the individual placement and support (IPS) model, shown to be effective for individuals with severe mental illness (Hoffmann et al., 2014). Similarly, for those suffering from long-term health conditions, additional support with accessing medical services may be of use.

## Appendix A

**Table A1.** Latent growth curve model comparison Table A1. long description.

	Model fit statistics			
	CFI	TFI	RMSEA	SRMR
Linear	0.939	0.945	0.059	0.084
**Quadratic**	**0.975**	**0.976**	**0.039**	**0.034**
Cubic	0.00	0.00	0.000	0.00

*Note:* The Quadratic Latent Growth Curve Model is bolded as this model was selected (as are the column headers).

**Table A2:** Growth parameter statistics for latent growth curve model Table A2 long description.

	Intercept		Linear		Quadratic	
**Overall mean (full sample)**	Mean	95% Cis	Mean	95% Cis	Mean	95% Cis
	1.909	(1.897 to 1.921)	−0.137	(−0.141 to −0.133)	0.006	(0.006 to 0.006)

## Appendix B

**Table B1:** Growth mixture modelling fit statistics Table B1 long description.

*k* model	AIC	BIC	Adj-BIC	VLMR-LRT (*p*=)	Entropy	% individuals in per class
*k* = 2	404081	404281	404202	<0.001	0.593	76/23
*k* = 3	403254	403487	403395	0.239	0.523	34/44/21
*k* = 4	402829	403094	402989	0.239	0.726	31/7/33/29
*k* = 5	402275	402572	402454	0.240	0.606	14/7/33/30/15
***k* = 6**	**401825**	**402154**	**402023**	**0.239**	**0.667**	**7/14/27/30/7/15**
*K* = 7	502334	502695	502552	0.239	0.665	4/15/7/31/12/7/23

*Note: k* = 6 model is bolded as this was the selected model (as are the column headers).

**Table B2.** Growth parameter statistics for final GMM models Table B2. long description.

	Intercept		Linear		Quadratic	
	Mean	95% Cis	Mean	95% Cis	Mean	95% Cis
Class 1	2.115	(2.054 to 2.176)	−0.246	(−0.273 to −0.219)	0.013	(0.011 to 0.015)
Class 2	2.867	(2.847 to 2.887)	−0.136	(−0.160 to −0.112)	0.008	(0.006 to 0.010)
Class 3	1.783	(1.693 to 1.873)	0.020	(−0.054 to 0.094)	0.002	(−0.006 to 0.010)
Class 4	2.934	(2.907 to 2.961)	−0.300	(−0.331 to −0.269)	0.01	(0.006 to 0.014)
Class 5	1.201	(1.168 to 1.234)	−0.049	(−0.071 to −0.027)	0.001	(−0.001 to 0.003)
Class 6	0.296	(0.265 to 0.327)	0.059	(0.034 to 0.084)	−0.005	(−0.007 to −0.003)

## Long descriptions

### Table 1. Long description

The table consists of three columns: Category, Variable, and Details and options (Notes).

1. Socio-demographics category includes:

- Gender: Self-reported as Male, Female, or Non-binary.

- Age: Continuous variable at referral.

- Ethnicity: Grouped into White, Mixed, Asian, Black, or Other.

- Sexual orientation: Binarized as Heterosexual or Non-Heterosexual.

- Index of multiple deprivations (I M D): Deciles 1 to 10 measuring relative deprivation in England.

- Employment status: Binarized as Employed (including students and homemakers) or Not Employed.

2. Psychological factors category includes:

- Generalized anxiety symptom severity: Assessed via G A D-7 scale.

- Personal functioning: Assessed via Work and Social Adjustment Scale.

3. Health factors category includes:

- Long-term health conditions: Self-reported as Yes or No.

- Psychotropic medication: Binarized as Taking or Not taking.

4. Service factors category includes:

- Service: Categorized by Trust 1, Trust 2, Trust 3, or Trust 4.

- Waiting time: Measured between referral and first assessment, and between first and second appointments.

5. Cohort factors category includes:

- Cohort: The year treatment began, entered as a continuous variable.

Navigate back to Table 1..

### Table 2. Long description

The table is divided into categorical demographics and continuous clinical characteristics.

Categorical Data (Characteristic, Category, n, percentage):

- Therapeutic treatment: C B T (18615, 82.34%), Counselling for depression (3410, 15.09%), Other/Missing (580, 2.57%).

- Gender: Female (15475, 68.46%), Male (7027, 31.09%), Nonbinary (33, 0.15%), Missing (70, 0.31%).

- Sexuality: Heterosexual (16586, 73.37%), Gay/Lesbian (728, 3.22%), Bisexual (669, 2.96%), Not sure (317, 1.40%), Missing/declined (4305, 19.04%).

- Ethnicity: White (13552, 59.95%), Mixed (1515, 6.70%), Asian (3055, 13.51%), Black (2657, 11.75%), Other (1081, 4.78%), Missing (745, 3.30%).

- Religion: No religion (5644, 24.97%), Christian (4277, 18.92%), Muslim (2048, 9.06%), Jewish (503, 2.23%), Hindu (496, 2.19%), Other (919, 4.07%), Missing (8718, 38.57%).

- Employment status: Employed (15947, 70.55%), Unemployed (5898, 26.09%), Missing (760, 3.36%).

- Taking medication: No (12574, 55.62%), Yes (8350, 36.94%), Missing (1681, 7.44%).

- Long-term condition: Yes (6143, 27.18%), No (10177, 45.02%), Missing (6285, 27.80%).

- Service: Trust 1 (8392, 37.12%), Trust 2 (4620, 20.44%), Trust 3 (4776, 21.13%), Trust 4 (4817, 21.31%).

Continuous Data (Characteristic, n, Mean, S D):

- Age at referral: 22605, 38.42, 13.22.

- P H Q 9 score: 22553, 16.44, 5.70.

- G A D 7 score: 22545, 13.52, 5.02.

- W S A S score: 22037, 20.36, 9.01.

- Weeks between referral and assessment: 22600, 4.63, 7.59.

- Weeks between referral and treatment: 21793, 13.35, 11.05.

- Number of attended sessions: 19124, 10.33, 4.92.

Navigate back to Table 2..

### Figure 1. Long description

The x-axis is labeled Session Number and ranges from 1 to 12. The y-axis is labeled P H Q 9 Item 1 and ranges from 0 to 3 in increments of 0.5. Two lines are plotted. The first line, representing Observed Data, is blue with circular markers and vertical error bars. It starts at approximately 1.95 at session 1 and follows a steady, slightly curved downward trend to approximately 1.1 at session 12. The second line, representing the Model, is light orange with black circular markers and vertical error bars. It starts slightly lower than the observed data at session 1, around 1.9, and follows a nearly identical downward trajectory, ending at approximately 1.05 at session 12. The two lines overlap significantly throughout the duration of the twelve sessions. A legend to the right of the plot identifies the blue line as Observed Data and the orange line with black dots as Model.

Navigate back to Figure 1..

### Figure 2. Long description

The X-axis is labeled Session Number and ranges from 1 to 12. The Y-axis is labeled P H Q 9 Item 1 and ranges from 0 to 3. Six distinct trajectory lines are plotted.

* Class 1 (27.2%): A dark blue line with circular markers starting at 2.1 and showing a steep exponential decay, leveling off at 1.0 by session 10.

* Class 2 (14.6%): A black dotted line with square markers starting at 2.9 and showing a gradual, slightly concave decrease to 2.3.

* Class 3 (6.6%): A black double-line with triangular markers starting at 1.8 and showing a steady linear increase to 2.2.

* Class 4 (14.2%): A solid light blue line starting at 2.9 and showing a constant linear decrease to 1.0.

* Class 5 (30.1%): A light peach line with circular markers starting at 1.2 and showing a gradual linear decrease to 0.8.

* Class 6 (7.3%): A thick black dashed line starting at 0.3, peaking slightly at 0.5 around session 7, and ending at 0.4.

Navigate back to Figure 2..

### Table 3. Long description

The table presents odds ratios with 95 percent confidence intervals and p-values for predictors across five anhedonia classes (C#1, C#2, C#3, C#4, and C#6) relative to Class 5. Significant results (p < 0.05) are bolded.

Research Aim 2 Predictors:

* Referral year: Significant for all classes, ranging from 0.924 to 1.009.

* Gender (Male vs Female): Significant for all; highest odds in C#6 (1.381) and lowest in C#4 (0.774).

* Sexual orientation (Non-heterosexual vs Heterosexual): Significant for C#2 (0.673), C#4 (1.093), and C#6 (1.259).

* G A D 7: Significant for all; highest in C#2 (1.321).

* W S A S 2, 3, and 4: Significant for all classes; generally showing higher odds for severe/moderate classes and lower odds (below 1.0) for C#6.

* W S A S 5: Significant for C#2, C#3, C#4, and C#6.

* Waiting time (referral to assessment): Significant only for C#4 (0.979).

* Waiting time (until second appointment): Significant for C#2 (1.007) and C#4 (0.992).

* Age: Significant for C#1 (1.007), C#2 (1.026), and C#4 (1.015).

* Medication (Taking vs Not taking): Significant for all; highest in C#2 (1.767).

* Long-term health conditions (Yes vs No): Significant for C#2 (1.393), C#3 (1.462), and C#6 (0.850).

* Employment (Unemployed vs Employed): Highly significant for all; notably high odds in C#2 (5.025) and C#3 (3.546).

* I M D Decile: Significant for C#2, C#3, C#4, and C#6.

* Ethnicity (relative to White): Black, Asian, and Other ethnicities show varying significant associations across all classes.

* Service (Trusts 2, 3, and 4 relative to Trust 1): Most associations are significant across all classes.

Research Aim 3 Predictors:

* Treatment type (C B T vs Counseling for depression): Significant for C#1 (1.183), C#2 (1.252), and C#4 (1.317).

Navigate back to Table 3..

### Table 4. Long description

The table consists of six columns. The first column lists baseline predictors, while columns two through six represent anhedonia trajectory classes: C number 1 (Moderate baseline, responder), C number 2 (Severe baseline, non-responder), C number 3 (Moderate baseline, non-responder), C number 4 (Severe baseline, responder), and C number 6 (Little-no baseline anhedonia symptoms). A plus symbol indicates increased likelihood and a minus symbol indicates reduced likelihood.

Key associations include:

* Referral year (Higher): plus for C number 1; minus for C number 2, C number 3, C number 4, and C number 6.

* Gender (male): plus for C number 1, C number 2, C number 3, and C number 4; minus for C number 6.

* G A D 7 (higher), W S A S 2 (home management), W S A S 3 (social leisure), and W S A S 4 (private leisure): plus for C number 1, C number 2, C number 3, and C number 4; minus for C number 6.

* Medication (taking): minus for C number 1, C number 2, C number 3, and C number 4; plus for C number 6.

* Employment (unemployed): plus for C number 1, C number 2, C number 3, and C number 4; minus for C number 6.

* Ethnicity (Asian): plus for C number 1, C number 2, C number 3, and C number 4; minus for C number 6.

* Treatment type (C B T): plus for C number 1, C number 2, and C number 4; blank for C number 3 and C number 6.

Navigate back to Table 4..

### Table 5. Long description

The table is divided into two main sections: Research Aim 2 and Research Aim 3.

Research Aim 2 predictors and their Class 4 O R, 95 percent C I, and p-values include:

* Referral year: 1.092 (1.087 to 1.096), p less than 0.001.

* Gender: Female is the reference 1.0; Male is 0.958 (0.939 to 0.978), p less than 0.001.

* Sexual orientation: Heterosexual is the reference 1.0; Non-heterosexual is 1.624 (1.357 to 1.943), p equals 0.001.

* G A D 7: 0.894 (0.862 to 0.927), p less than 0.001.

* W S A S 2: 0.944 (0.896 to 0.994), p equals 0.021.

* W S A S 3: 0.899 (0.835 to 0.967), p equals 0.001.

* W S A S 4: 0.969 (0.918 to 1.024), p equals 0.243.

* W S A S 5: 0.977 (0.927 to 1.031), p equals 0.371.

* Waiting time (referral to assessment): 0.985 (0.970 to 1.000), p equals 0.059.

* Waiting time (until second appointment): 0.985 (0.977 to 0.994), p equals 0.003.

* Age: 0.990 (0.982 to 0.999), p equals 0.012.

* Medication: Not taking is the reference 1.0; Taking is 0.924 (0.877 to 0.974), p equals 0.002.

* Long-term health conditions: Yes is 0.762 (0.684 to 0.848), p less than 0.001; No is the reference 1.0.

* Employment: Employed is the reference 1.0; Unemployed is 0.344 (0.330 to 0.358), p less than 0.001.

* I M D decile: 1.038 (0.988 to 1.091), p equals 0.151.

* Ethnicity: White is 0; Black is 1.804 (1.692 to 1.922), p less than 0.001; Mixed is 1.389 (1.275 to 1.514), p less than 0.001; Asian is 1.107 (1.046 to 1.172), p equals 0.001; Other is 1.201 (1.108 to 1.302), p less than 0.001.

* Service: Trust 1 is 0; Trust 2 is 0.730 (0.710 to 0.751), p less than 0.001; Trust 3 is 0.577 (0.560 to 0.595), p less than 0.001; Trust 4 is 0.783 (0.764 to 0.802), p less than 0.001.

Research Aim 3 predictors include:

* Treatment type: C B T is 0.950 (0.724 to 1.247), p equals 0.712; Counselling for depression is the reference 1.0.

Navigate back to Table 5..

### Table 6. Long description

The table is divided into Research Aim 2 and Research Aim 3. Values are reported as Odds Ratio O R, 95 percent Confidence Interval C I, and p-value. Significant results with p less than 0.05 are in bold.

Research Aim 2 predictors:

* Referral year: 1.061 (1.056 to 1.066), p less than 0.001.

* Gender: Female is reference 1.0; Male is 1.039 (1.010 to 1.070), p equals 0.011.

* Sexual orientation: Heterosexual is reference 1.0; Non-heterosexual is 0.955 (0.785 to 1.160), p equals 0.628.

* G A D 7: 0.979 (0.955 to 1.003), p equals 0.077.

* W S A S 2: 0.960 (0.908 to 1.016), p equals 0.145.

* W S A S 3: 0.997 (0.969 to 1.027), p equals 0.841.

* W S A S 4: 0.984 (0.957 to 1.012), p equals 0.249.

* W S A S 5: 0.948 (0.911 to 0.986), p equals 0.005.

* Waiting time referral to assessment: 0.995 (0.980 to 1.010), p equals 0.531.

* Waiting time until second appointment: 0.990 (0.981 to 0.999), p equals 0.012.

* Age: 1.003 (0.993 to 1.013), p equals 0.549.

* Medication: Not taking is reference 1.0; Taking is 0.739 (0.689 to 0.793), p less than 0.001.

* Long-term health conditions: Yes is 0.672 (0.580 to 0.778), p less than 0.001; No is reference 1.0.

* Employment: Employed is reference 1.0; Unemployed is 0.364 (0.346 to 0.384), p less than 0.001.

* I M D decile: 1.070 (1.042 to 1.098), p less than 0.001.

* Ethnicity: White is reference 0; Black is 0.962 (0.907 to 1.020), p equals 0.182; Mixed is 1.179 (1.066 to 1.304), p equals 0.007; Asian is 0.813 (0.762 to 0.869), p less than 0.001; Other is 0.424 (0.392 to 0.459), p less than 0.001.

* Service: Trust 1 is reference 0; Trust 2 is 0.717 (0.688 to 0.747), p less than 0.001; Trust 3 is 0.860 (0.835 to 0.886), p less than 0.001; Trust 4 is 1.116 (1.088 to 1.143), p less than 0.001.

Research Aim 3 predictors:

* Treatment type: C B T is 0.916 (0.664 to 1.264), p equals 0.594; Counselling for depression is reference 1.0.

Navigate back to Table 6..

### Table A1. Long description

The table presents four model fit statistics: C F I, T F I, R M S E A, and S R M R.

* Linear model: C F I is 0.939, T F I is 0.945, R M S E A is 0.059, and S R M R is 0.084.

* Quadratic model: This row is bolded, indicating the best fit. C F I is 0.975, T F I is 0.976, R M S E A is 0.039, and S R M R is 0.034.

* Cubic model: All statistics are listed as 0.00 or 0.000.

Navigate back to Table A1..

### Table A2 Long description

The table presents data for the Overall mean of a full sample across three growth parameters.

1. Intercept: The Mean is 1.909 with 95 percent C I s ranging from 1.897 to 1.921.

2. Linear: The Mean is minus 0.137 with 95 percent C I s ranging from minus 0.141 to minus 0.133.

3. Quadratic: The Mean is 0.006 with 95 percent C I s ranging from 0.006 to 0.006.

Navigate back to Table A2.

### Table B1 Long description

The table consists of seven columns: k model, A I C, B I C, Adj-B I C, V L M R-L R T (p equals), Entropy, and percentage of individuals per class.

* k equals 2: A I C 404081, B I C 404281, Adj-B I C 404202, p less than 0.001, Entropy 0.593, Class distribution 76/23.

* k equals 3: A I C 403254, B I C 403487, Adj-B I C 403395, p 0.239, Entropy 0.523, Class distribution 34/44/21.

* k equals 4: A I C 402829, B I C 403094, Adj-B I C 402989, p 0.239, Entropy 0.726, Class distribution 31/7/33/29.

* k equals 5: A I C 402275, B I C 402572, Adj-B I C 402454, p 0.240, Entropy 0.606, Class distribution 14/7/33/30/15.

* k equals 6 (bolded): A I C 401825, B I C 402154, Adj-B I C 402023, p 0.239, Entropy 0.667, Class distribution 7/14/27/30/7/15.

* k equals 7: A I C 502334, B I C 502695, Adj-B I C 502552, p 0.239, Entropy 0.665, Class distribution 4/15/7/31/12/7/23.

Navigate back to Table B1.

### Table B2. Long description

The table is organized into seven columns. The first column lists the classes, and the subsequent columns are paired under three headers: Intercept, Linear, and Quadratic. Each pair includes a Mean and 95 percent C I s (Confidence Intervals).

* Class 1: Intercept Mean 2.115 (2.054 to 2.176); Linear Mean minus 0.246 (minus 0.273 to minus 0.219); Quadratic Mean 0.013 (0.011 to 0.015).

* Class 2: Intercept Mean 2.867 (2.847 to 2.887); Linear Mean minus 0.136 (minus 0.160 to minus 0.112); Quadratic Mean 0.008 (0.006 to 0.010).

* Class 3: Intercept Mean 1.783 (1.693 to 1.873); Linear Mean 0.020 (minus 0.054 to 0.094); Quadratic Mean 0.002 (minus 0.006 to 0.010).

* Class 4: Intercept Mean 2.934 (2.907 to 2.961); Linear Mean minus 0.300 (minus 0.331 to minus 0.269); Quadratic Mean 0.01 (0.006 to 0.014).

* Class 5: Intercept Mean 1.201 (1.168 to 1.234); Linear Mean minus 0.049 (minus 0.071 to minus 0.027); Quadratic Mean 0.001 (minus 0.001 to 0.003).

* Class 6: Intercept Mean 0.296 (0.265 to 0.327); Linear Mean 0.059 (0.034 to 0.084); Quadratic Mean minus 0.005 (minus 0.007 to minus 0.003).

Navigate back to Table B2..
